# The Impact of Excluding Food Guarding from a Standardized Behavioral Canine Assessment in Animal Shelters

**DOI:** 10.3390/ani8020027

**Published:** 2018-02-08

**Authors:** Heather Mohan-Gibbons, Emily D. Dolan, Pamela Reid, Margaret R. Slater, Hugh Mulligan, Emily Weiss

**Affiliations:** 1 Strategy, Research and Development, American Society for the Prevention of Cruelty to Animals (ASPCA^®^), New York, NY 10018, USA; heather.mohan-gibbons@aspca.org (H.M.-G.); emily.dolan@aspca.org (E.D.D.); margaret.slater@aspca.org (M.R.S.); hugh.mulligan@aspca.org (H.M.); 2 Anti-Cruelty Behavior Team, Anti-Cruelty Group, American Society for the Prevention of Cruelty to Animals (ASPCA^®^), New York, NY 10018, USA; Pam.Reid@aspca.org; 3 Equine Welfare, Anti-Cruelty Group, American Society for the Prevention of Cruelty to Animals (ASPCA^®^), New York, NY 10018, USA

**Keywords:** food guarding, shelter assessment, dogs, aggression, animal shelter, euthanasia, SAFER, behavior assessments, ASPCA

## Abstract

**Simple Summary:**

Recent research has called into question the value of the food guarding assessment as a predictive tool for determining the safety of shelter dogs. This study examined the effect of eliminating the food guarding assessment in nine U.S. animal shelters. It was found that when the food guarding assessment was removed, bites or other injuries to staff or adopters did not increase. However, dogs exhibiting food guarding behavior were less likely to be adopted, had a longer shelter stay, and were more likely to be euthanized than dogs in the general population. Based on previous research and this study’s findings, the authors recommend that shelters discontinue the food guarding assessment.

**Abstract:**

Many shelters euthanize or restrict adoptions for dogs that exhibit food guarding while in the animal shelter. However, previous research showed that only half the dogs exhibiting food guarding during an assessment food guard in the home. So, dogs are often misidentified as future food guarders during shelter assessments. We examined the impact of shelters omitting food guarding assessments. Nine shelters conducted a two-month baseline period of assessing for food guarding followed by a two-month investigative period during which they omitted the food guarding assessment. Dogs that guarded their food during a standardized assessment were less likely to be adopted, had a longer shelter stay, and were more likely to be euthanized. When the shelters stopped assessing for food guarding, there was no significant difference in the rate of returns of food guarding dogs, even though more dogs were adopted because fewer were identified with food guarding behavior. Additionally, the number of injuries to staff, volunteers, and adopters was low (104 incidents from a total of 14,180 dogs) and did not change when the food guarding assessment was omitted. These results support a recommendation that shelters discontinue the food guarding assessment.

## 1. Introduction

Food guarding (FG) is a natural behavior for Canids, yet can result in adverse consequences when domestic dogs exhibit this behavior in animal shelters. FG can occur when a dog has a consumable item that it wants to retain. Guarding behaviors, such as growling and snapping towards a perceived competitor, can be seen in puppies as young as two to three weeks of age [1]. Most, if not all, standardized behavior assessments currently used in shelters [2,3,4] include an evaluation of FG behavior. These assessments typically evaluate the extent of FG by using a fake hand, made of rubber or plastic on a dowel, to pull the food bowl away from the dog and touch their cheek while eating. Different assessments vary the time and degree of contact between the dog and the fake hand. In 2005, The American Society for the Prevention of Cruelty to Animals^^®^^ (ASPCA^^®^^) [5] conducted a survey of US animal shelters. Eighty-nine percent of responding organizations reported they conducted a behavior assessment in their shelter and almost all assessed for guarding around a food bowl. For the sake of concision in this manuscript, the specific FG component of the behavior assessment is referred to as the FG item.

Two recent studies indicated that assessing for FG in the shelter was a poor predictor of FG behavior in the home. Marder et al. [6] looked at 97 dogs adopted from an animal shelter and compared their FG behavior during assessment and in-home. All dogs were assessed using Match-Up II Shelter Dog Rehoming program [4]. Twenty (21%) dogs exhibited FG in the assessment and of those, 11 (55%) showed FG in-home. Interestingly, of the 77 dogs that did not display FG during the assessment, 17 (22%) did display FG in-home. Whether or not dogs guarded food during the shelter assessment had little bearing on the FG behavior they displayed in-home.

The second study [5] followed 96 dogs that exhibited FG during the Safety Assessment for Evaluating Rehoming (SAFER™) assessment [2]. Dogs that exhibited FG were allowed free-access to dry dog food in their kennel, and then subsequently adopted into homes. Three weeks after adoption, only one of the 96 dogs was reported to exhibit FG behavior in the home. By three months, no dogs were reported showing FG behavior in-home. The discrepancy between these two studies in the rate of FG in-home could be a result of the free feeding regimen the dogs experienced while in the shelter, substantially different durations of follow-up, support given to adopters, fundamental differences in the shelter dog populations, or other unidentified variables. However, the conclusions regarding the inability of the FG item to reliably predict FG in-home is consistent across the two studies.

Both of these studies call into question the value of assessing FG while the dog is in the shelter. It appears that dogs showing FG guarding during the assessment are no more or less likely to exhibit FG in a home. Equally as important, the Marder study [6] found that adopters did not perceive FG behavior as a problem in keeping their dog, which suggests that standardized FG assessments may be unnecessary and irrelevant.

In 2016, Patronek and Bradley [7] considered the usefulness of standardized behavior assessments in shelters and noted that “reliably predicting problematic behaviors in future adoptive homes is vanishingly unlikely.” They argued that if a dog bit or threatened to bite during a standardized assessment that whether or not the dog would do so in an adoptive home “would be at best, not much better than flipping a coin”. Although the authors were referring to aggression assessments in general, and not just the FG portion of the assessment, their primary argument is that standardized assessments result in an unacceptably high incidence of false positives (which are those dogs that exhibit aggression during the assessment and not in-home).

A high rate of false positives is particularly alarming when it comes to FG, because FG was one of the top reasons shelter staff reported a dog would be deemed unadoptable [5] and euthanized [6]. Even when a shelter chose to not euthanize dogs with FG behavior, those dogs were more likely to have adoption restrictions (such as experienced owners only or families without children) thereby reducing the pool of potential adopters for the dogs [6].

Since the publication of the Marder et al. [6] and the Mohan-Gibbons et al. [5] studies, members of the animal welfare field have shared their interest and concerns with the ASPCA^^®^^ about omitting the FG item from their assessment process. Their primary concerns were that injuries to staff or adopters would increase and that dogs would be returned more often. Thus, the primary goals of this study were to compare the outcomes, injuries, and returns after the FG item was removed from the assessment.

## 2. Materials and Methods

### 2.1. Inclusion Criteria

In order to be included in this study, shelters had to already be using a standardized behavior assessment that included a FG item and be willing to discontinue the FG item for the investigation phase. The FG item involved presenting the dog with a bowl of food and then using a fake hand on a dowel to touch the dog and the bowl while the dog was eating the food. Only FG toward people was addressed; FG directed towards other dogs was not a part of any standardized assessment in this study. Any dog exhibiting FG was included, regardless of breed or age (as long as they were at least 6 months old, per assessment guidelines). Shelters refrained from making any other program or adoption changes during the study that would have affected the research.

### 2.2. Recruitment of Shelters

Animal shelters were recruited for this study through a convenience sample. The authors had prior relationships with some of the shelters and spoke directly with the staff to ask for their participation. Other organizations volunteered themselves for the study following a presentation on guarding behaviors in the shelter given by Drs. Emily Weiss and Amy Marder at the Society of Animal Welfare Associations conference in 2014. The authors also sought recommendations from other leaders in the field to identify organizations that might be interested in participating. Any organization that expressed interest in participating was sent an email providing a summary of the project.

A total of 11 shelters enrolled in the study. In the first month, 2 shelters were excluded: 1 shelter pre-screened dogs for FG as part of their relocation program, rendering them inadmissible and the other shelter withdrew because they felt they could not meet the data collection requirements.

The shelters were all non-profit humane societies except for one large municipal animal control organization. These shelters were located in seven states: Colorado, Florida, Mississippi, New York, North Carolina, and South Carolina. There was one shelter in each of these states except for Florida and New York, which each had two. Seven shelters used the SAFER™ assessment and two shelters used their own standardized assessment.

### 2.3. Protocol

Nine shelters completed the 5-month study from 1 September 2015 to 31 January 2016. In the first 2 months (baseline period), shelters were instructed to continue with their regular process for assessing behavior, then they were to omit the FG item for the next 2 months (investigation phase). In the investigation phase, shelters were advised to conduct all other components of their assessment (minus the FG item) and to proceed with adoptions as per normal. A data collection form was issued to each shelter to collect the following information for each month of the baseline phase and the investigation phase for the entire dog population: (1) total intake, (2) total returns of dogs adopted within the past 30 days, (3) average length of stay, (4) total number of dogs adopted, transferred, and euthanized; and the total number of bites and injuries (5) observed while in-shelter and (6) reported in-home post-adoption. Those data were also collected for the dogs that were classified as food guarders in both the baseline and investigation phases. During the last month of the study, in January, the only data collected were the number of returns and reports of bites or injuries that occurred in-home (#2 and #6 above).

Bites and injuries were collected as a single category, defined as any type of injury to a human, in any context, while in the shelter or after adoption. This information was gleaned from reports in the shelter and from conversations when owners contacted the shelter to discuss their dog post-adoption. Shelters were asked to report all incidents for any reason and, as a result, an incident could occur during any interaction with the dog and were not restricted to interactions around food. For example, a dog in the FG group may have caused harm by pulling someone off their feet while on a walk was still counted as an injury, even though no food was involved. This ensured a conservative approach and that all incidents were captured for this research.

On a monthly basis, completed forms were collected from each shelter by email. At the end of the study, the authors conducted an informal conference call with all of the shelters to learn more about their experiences during the study.

### 2.4. Identification of FG

Shelters were instructed to identify FG in one of three ways: (1) during the FG item of the assessment in the baseline phase, (2) from interviews conducted with the person who brought the animal to shelter, or (3) through observations made by staff, volunteers, and potential adopters while the dog was housed at the shelter (separate from the assessment). As noted in Section 2.3, the FG item was performed only during the baseline phase but the other ways of identifying FG were utilized over the entire study period. Once staff saw a dog exhibiting FG behavior, they recorded on the standardized data collection form how that behavior was identified.

The researchers provided shelters with descriptions of FG to enable them to categorize the level of severity of the guarding behavior. These descriptions were labeled as mild, moderate or severe (Appendix A). The behaviors could be seen in various contexts including during the assessment, in their kennel, or reported from the owner. Staff documented the level of severity for each dog regardless of how the behavior was identified.

### 2.5. Grants

Once enrolled in the study, each shelter was issued a $2500 grant to support the work needed to complete the study. Grant funds were used to train staff, create literature on FG for adopters and staff, start a behavior hotline, or recruit shelter volunteers.

### 2.6. Statistical Analysis

Characteristics of the groups were described using frequencies and percentages calculated with Microsoft Excel. Chi-square tests were performed to compare the baseline and investigation phase rates of bites/injuries and returns for the entire general dog population, as well as specifically for the group of FG dogs. Chi-square was also used to compare the percentage of food guarders and non-food-guarders euthanized during the four-month study period. StataSE 13.1 (StataCorp LP, College Station, TX, USA) was used for these analyses, and *p* < 0.05 was considered to be statistically significant.

## 3. Results

### 3.1. Population

A total of 14,180 dogs entered the nine shelters across the four-month study period. The highest intake at a participating shelter was 3247 dogs and the lowest intake was 596 dogs. During the baseline phase, 7112 dogs entered the shelters and 7068 dogs entered during the investigation phase. During the baseline phase, only 49% of the dogs were assessed upon intake. The other dogs were returned to their owners before an assessment could be completed or were considered adoptable without being assessed, due to their desirable appearance, size, or breed.

**Figure 1:**
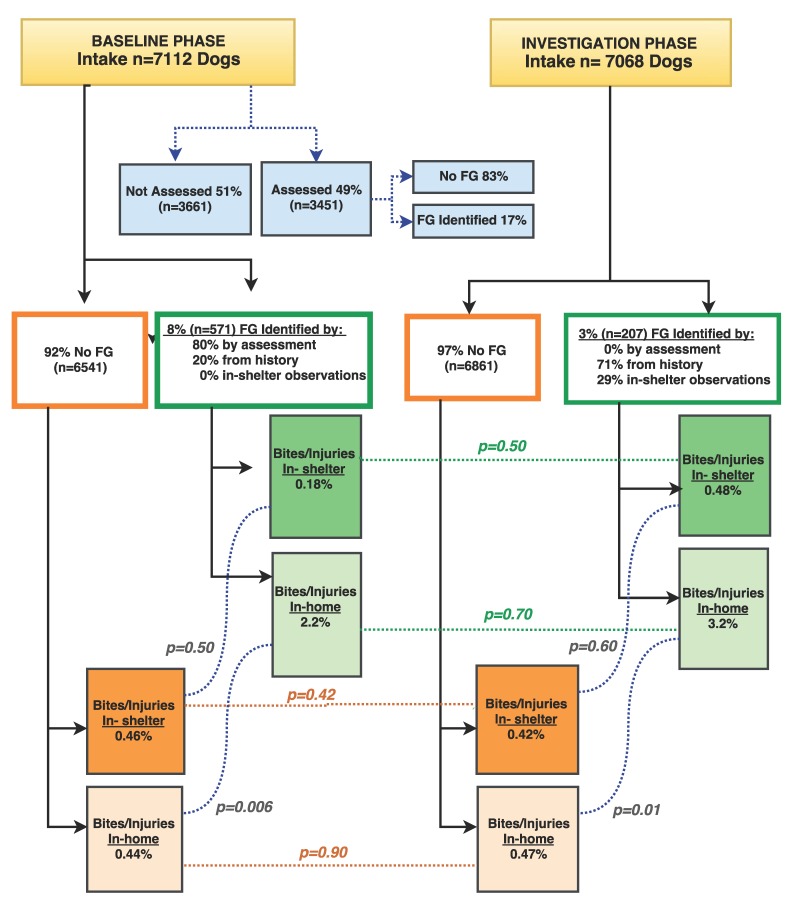
Total intake of dogs between the two study phases: percentage of dogs who were assessed, those dogs identified with food guarding behavior, and incidence of injury/bites in each phase. Non-FG groups are in orange and food guarding (FG) groups in green, with corresponding *p*-values displayed between the baseline and investigative phases. In baseline phase, there are blue boxes and dotted lines that show the percentage of dogs that had the standardized shelter assessment and of those assessed, how many were identified with FG. This was added so the reader can visualize the large percentage of dogs that did not have a standardize assessment performed and to clarify the two ways a shelter can arrive at incidence of FG (17% is only of those assessed while 8% is all intake).

Over the whole study period, 5% (778/14,180) of the dogs either exhibited FG or had a history of FG. Further details of which dogs were identified with FG during each phase can be found in Figure 1.

### 3.2. Risk of Injury

Figure 1 shows total intake of dogs between the two study phases and color groupings for those who displayed FG (green) vs non-FG (orange). The number of bites/injuries was low in both phases. FG dogs, either in the shelter or in the home, were no more likely to inflict injuries once the assessment item was stopped. A within-phase study analysis was also conducted. In the home, adopters were significantly more likely to report bites/injuries from dogs in the FG group compared to the non-FG group, in both baseline (*p* = 0.006) and investigate stages (*p* = 0.01). In the home, there were only 8 reported bites/injuries for the FG group and 35 for the non-FG group. In the shelter, there was no association of bites/injuries related to the FG or non-FG groups, in baseline (*p* = 0.5) or investigative phases (*p* = 0.6).

### 3.3. Outcomes for FG Dogs

Out of the 14,180 dogs entering the shelter, 14% (2051/14,180) were still in the shelter at the termination of the study and could not be assigned an outcome. Over the course of the study, 56% (7961/14,180) of all dogs were adopted and 18% (2576/14,180) were transferred to other organizations for adoption. Fifty-four percent of dogs (3867/7112) were adopted during the baseline phase and 58% (4094/7068) during the investigation phase. Adoption of FG dogs was 39% in the baseline (223/571) and 45% the investigation phase (93/207) (*p* = 0.14, X^2^ = 2.17, 1 df).

Across both study phases, dogs that showed FG behavior stayed in the shelter longer, a mean of 13.6 days (range = 4.7–44 days), than the general dog population, a mean of 9.9 days (range = 3.6–20 days).

A total of 11.2% of dogs (1592/14,180) were euthanized during the study. Significantly more FG dogs (15.4%, 120/778) were euthanized than non-FG dogs (10.9%, 1472/13,402), *p* < 0.001, X^2^ = 0.09, 1 df. Of the 120 dogs euthanized in the FG group, 74% (*n* = 89) had behavior listed as a reason for euthanasia and 45% of those had FG listed as a reason for euthanasia. The other 55% were euthanized for other behavioral issues such as aggression toward other animals or people, high arousal, or problematic behavior in the kennel.

#### Degree of FG and Impact

If dogs exhibited FG in-shelter, the majority of the time it was mild in severity (Table 1). Another key finding is the percentage of dogs identified with severe FG was unchanged, even once the FG item was omitted.

**Figure 2:**
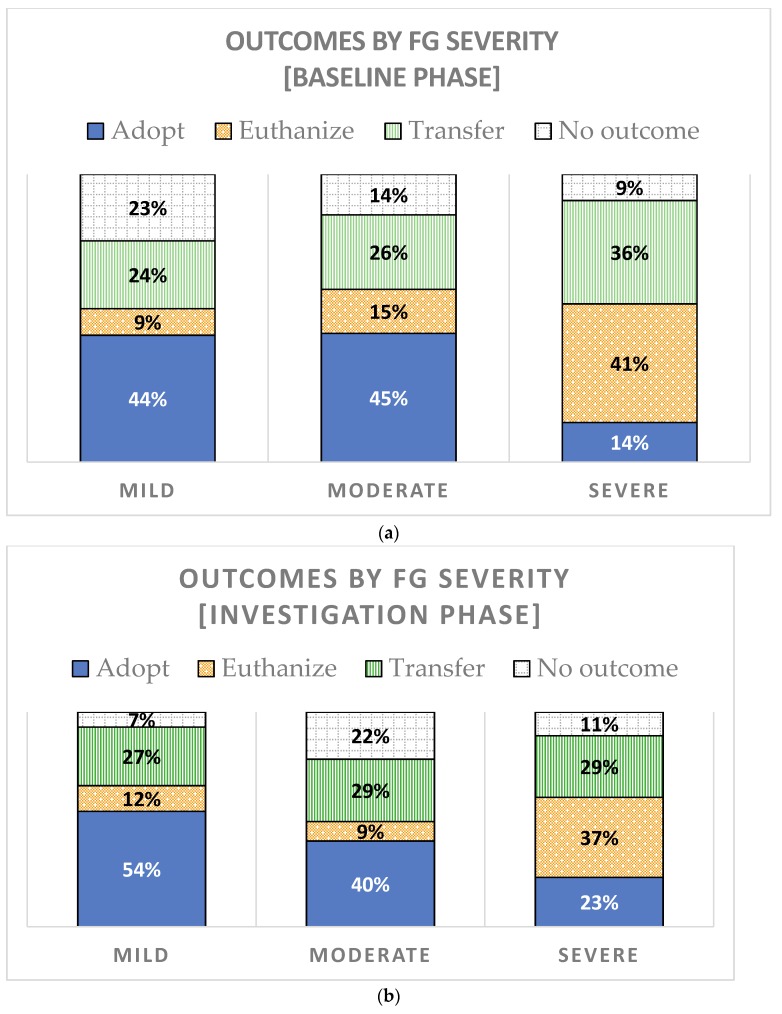
Outcomes of the FG dogs, by severity of FG behavior, are shown for the (**a**) Baseline phase (**b**) Investigation phase. Some dogs in each severity category did not have an outcome by the end of the study period (no outcome). In the baseline, 20% of FG was identified by history and 80% by the assessment. During the investigation phase, 71% of FG was identified by history and 29% from shelter observations.

Figure 2 shows outcomes of adoption, euthanasia, and transfers, categorized by the severity of FG. During both baseline and investigation phases, dogs with severe FG behavior were less likely to be adopted and more likely to be euthanized than moderate or mild food guarders.

### 3.4. Returns

Dogs in the FG group were no more likely to be returned (*p* = 0.7, X^2^ = 0.11, 1 df) in the investigation phase (11%, 10/93) than they were during the baseline phase (9%, 21/223). There were significantly more returns of all dogs (*p* < 0.001, X^2^ = 18.33, 1 df) during the investigation phase (13%, 526/4094) than during the baseline phase (10%, 379/3867). However, even though statistically significant, a difference of 3% in returns is too small to be meaningful to the field.

## 4. Discussion

This study documented the impact of omitting the FG item from standardized shelter behavior assessments. During the baseline phase, 8% of dogs were classified as food guarders. During the investigation phase, when dogs were no longer assessed for FG, only 3% were classified as food guarders. Other studies [5,6] reported higher percentages of FG (14–21%), however they looked at the number of FG dogs based on the number assessed, not on overall intake numbers. If this study were to calculate FG dogs based on those assessed (which could only be done during baseline phase), the percentage aligns with the other studies (16.5%). However, since many shelters assess less than half their dog population, these reported percentages likely over-represent the prevalence of FG behavior in the shelter dog population.

When designing this study, it was not anticipated that such a large number of dogs would not be assessed during baseline. While it is not known what each individual staff person did within each shelter (relative to the dogs not assessed), shelters did not always assess dogs who were easily adoptable (i.e., healthy, small, obviously social, and friendly dogs). It is not clear how much bias this brings and in what direction the bias would be, due to the lack of assessments among a subset of the population of dogs. One key factor used to improve validity was each shelter was its own baseline. This study was designed to be applied research in functioning shelters, constrained by shelter realities. 

Since this was a convenience sample, there could be a bias in those shelters that chose to participate. The shelters varied in size (number of dogs and staff), one was a municipal shelter while most were non-profit organizations, and they varied in their location around the country. We deliberately aimed for a large sample size from a variety of shelters to minimize biases in the results and improve external validity.

The safety of staff, volunteers, and adopters was identified as a critical concern when shelters considered removing the FG item from their assessments. However, few bites or injuries were reported during this study, whether or not dogs were assessed for FG. It is interesting that the rate of bites and injuries was so low, even in the baseline phase, when a significant number of dogs were not assessed. This further confirms the conclusion that testing for FG does not improve safety in a shelter setting.

Reported injuries in the home did not increase once the FG item was stopped, however there was a higher percentage of injuries by FG dogs compared to the non-FG dog group. However, there was a low prevalence of bites/injuries in the home for all dogs (Figure 1) and the reporting included all bites and injuries, even when not involving food. Since many shelters advised adopters that these dogs might FG in the home, it is possible that new owners were more likely to report bites or injuries thus biasing the numbers. It is plausible that dogs who FG in the shelter may also have other behavior problems that make them more likely to cause injuries post-adoption, but this study did not yield that data.

Another limitation of this study, which was also beyond the scope, was a lack of systematic follow-up in the home, like the protocol used in the Mohan-Gibbons study [5]. As a result, we could not ask for more information on the situations where the bite or injury occurred in the home. We did not ask shelters to separate actual bites from other types of injuries, nor did we ask for detail about the incidences. Although collecting that information would have provided more behavioral context, the concern was it would introduce unwanted bias because staff and adopters would need to correctly interpret every situation if FG was present or not. By tracking all incidences of bites and other injuries, our conclusions are more conservative.

In this study, dogs with food guarding behavior stayed in the shelter longer than the general population and were more likely to be euthanized. An earlier survey [5] found that FG behavior was one of the top reasons why shelters did not place dogs into adoption. In this study, while only 15.4% of FG dogs were euthanized, over half of these were euthanized for behavior concerns unrelated to FG (i.e., medical problems and other severe behavior challenges).

Most of the dogs that showed FG behavior in both the baseline and investigation phases were classified as mild FG. In the investigation phase, even though fewer dogs were identified with food guarding behavior, the percentage of dogs classified as severe remained unchanged (17% in both phases). Thus, it appears that dogs exhibiting more serious FG behavior are likely to be identified without using a standardized assessment. Relinquishing owners may report that their dog displays FG when it is more serious and staff may be more likely to observe severe guarding behavior during daily interactions in the shelter. This confirms the assertion made by Patronek & Bradley [7] that more serious forms of aggression are highly likely to be identified without putting the dog through a behavior assessment.

The animal welfare community had expressed concerns that returns to the shelter would increase if the FG item was eliminated. Our study showed that return rates for FG dogs were not significantly different from the baseline phase, however the return rate for the general population was. Even though those returns presumably included dogs that guarded food in the home, a difference of 3% is not large enough to be meaningful in the context of shelter returns. The statistical significance is driven by the large number of dogs adopted from the nine shelters. A dog returned from the FG group may not have been returned for food guarding. The reasons given for returns were insufficient to provide any level of detail. It is challenging to gather quality data when those data are based on owners relaying details to shelter personnel who then capture that information into restrictive shelter software. Future work could consider learning more about the reasons for dogs being returned to the shelter.

If shelters wish to further reduce returns, we recommend that they have conversations with all adopters about normal canine behavior, such as FG, and that any dog might exhibit this behavior in the home. Staff should encourage adopters to call the shelter if they encounter problems and need help. Other research has shown that when pet owners are given realistic expectations, they are more likely to keep their pets [8]. Perhaps providing adopters with basic information about FG and offering post-adoption support will prove to be a key to successful adoptions for this at-risk population.

Shelters did not make large organizational changes during the course of the study. Shelter staff was aware when the FG was stopped. We encouraged shelters to talk with owners relinquishing dogs to obtain a history of FG, and some shelters changed their intake forms to reflect this. We encouraged adoption counselors to tell potential adopters that any dog might guard their food and some added this information to their adoption paperwork. Many shelters reported that staff became more diligent about recognizing and reporting FG behavior in the investigative phase because the dogs were not going to be assessed for FG. One shelter even uncovered that staff would not report observations of a dog guarding food in the kennel (during the baseline phase) because they believed “the behavior staff would catch it in the FG item during the assessment”. It is crucial for shelters to establish a clear communication system for staff and volunteers to share pertinent behavior information about the dogs in their care, especially when considering omitting the FG item from their assessment.

In previous research, a significant number of dogs that were observed guarding food during the assessment did not exhibit this behavior in the home [5,6]. These “false positive” dogs are more likely to stay longer in the shelter and to be euthanized. In addition, some dogs do not guard food while in the shelter but will at home. These “false negative” dogs are often incorrectly deemed as “safe” by shelter staff and sent home with adopters who are not adequately prepared to encounter this behavior. Any dog can exhibit food guarding behavior. Our research, paired with previous research referenced in this manuscript, substantiate that the existence of food guarding behavior in the shelter does not always predict the same behavior in the home. The incidence of injury to shelter staff and adopters is low and assessing dogs for FG does not further reduce the already low injury rate. 

## 5. Conclusions and Recommendations

Previous research suggested that many dogs exhibiting FG in the shelter can be safely placed into homes because FG is often not exhibited in the home nor was it a problem for adopters when it was seen. In light of the current results, the authors recommend that shelters need not conduct the FG item of a standardized assessment because it results in a longer stay in the shelter, increased likelihood of euthanasia, and the incidence of false positives is likely unacceptably high. FG was not prevalent in the participating nine shelters and, of the dogs that did exhibit the behavior, most showed only mild FG behavior. Once the FG item was omitted, more of those dogs were adopted and fewer were euthanized. Severe FG behavior was found regardless of the assessment. Discontinuing the FG assessment item did not result in an increase in injuries to shelter staff or adopters, and there was no meaningful change in returns post-adoption.

Our recommendation is for shelters to collect as much information about the dog from all sources (such as prior owner and staff observations) to best support each dog in finding a home. For example, if a dog shows guarding behavior over his food while in the kennel, the shelter should have procedures to ensure staff are trained so that they can stay safe while interacting with the dog. However, they should not assume the dog will show this behavior in his new home. Shelters should not ignore FG behavior. Rather, staff should be transparent with adopters about what they know from prior history and behavior in-shelter. Staff should set adopters up for success by explaining that FG is a normal behavior for dogs, it has a low occurrence, and if seen, they should contact the shelter promptly for assistance. There are techniques for managing and modifying FG behavior in the home when the owners are both concerned as well as able to implement these techniques.

## Figures and Tables

**Table 1:** Severity of Food guarding (FG) behavior identified in all FG dogs comparing the baseline (8%, 571/7112) and investigation (3%, 207/7068) phases. There was an overall significant difference in the categories of severity between the baseline and investigative phase (*p* = 0.001, X^2^ = 14.86, 2 df). In the baseline, 20% of FG was identified by history and 80% by the assessment. During the investigation phase, 71% of FG was identified by history and 29% from shelter observations.

Severity of FG Behavior	Baseline		Investigation	
	*N*	%	*N*	%
**Total Dogs with FG Behavior**	**571**	**8%**	**207**	**3%**
Mild	391	68%	117	57%
Moderate	85	15%	55	27%
Severe	95	17%	35	17%
**Total**	571	100%	207	100%

**Figure A1:**
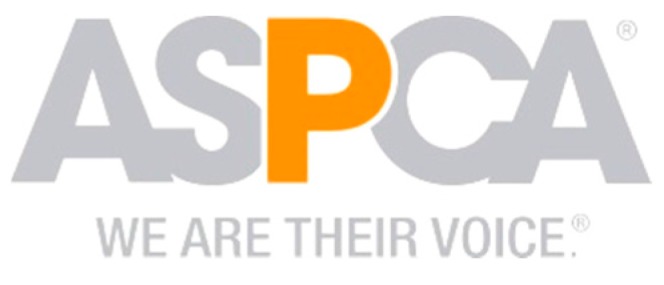
Behavioral Categories for Food Guarding.

## Appendix A

Below are three categories with descriptions of guarding behaviors that may be observed during the food portion of an assessment. Please choose the broad category that is the best fit for each dog. Vocal behaviors, such as growling or barking, are not listed, as they can be observed in any category.

### Appendix A.1. Mild

Dog’s head, neck and/or body become stiff/rigid but the dog does not escalate to Moderate or Severe categories. May show one or more of these behaviors:

- eats more quickly as food assessment progresses
- holds a crouched position over bowl when person is near
- moves body and/or head to block the bowl from the assessor’s approach or the fake hand
- momentarily freezes or stops eating (without lifting head from bowl) when assessor approaches or reaches with fake hand

### Appendix A.2. Moderate

The guarding behavior remains in or near the food bowl. Dogs may show any of the behaviors in the Mild category plus one or more of these behaviors:

- may lift lips/bare teeth
- bites in the direction of the food or at the fake hand while head is in the bowl and/or while touching the dog’s cheek with the fake hand or person is near
- although the dog may bite the fake hand, the dog does not lift his head from the bowl to bite toward the assess-a-hand or pursue the hand as it is retracted

### Appendix A.3. Severe

The guarding behavior is directed towards the assessor or handler. Dogs may show any of the Mild or Moderate behaviors, plus at least one of the following:

- multiple bites to the fake hand and/or bites that move up the fake hand
- leaves the bowl to bite the fake hand (either while the hand is advancing towards the bowl or as it is withdrawn)
- any attempts to lunge, snap, or bite towards the person’s arm or body or the handler during the food bowl item
- dogs that exhibit guarding behavior either before or after the food guarding assessment (for example, the bowl is placed down and the dog immediately shows any guarding behavior before assessor has attempted their initial approach to the bowl or dogs that continue to guard the area after the food bowl is removed).
